# Le Trouble de la Personnalité Limite à L’ère de TikTok : Exploration Qualitative des Discours D’internautes: Borderline Personality Disorder in the Age of TikTok: A Qualitative Exploration of Internet Users’ Comments

**DOI:** 10.1177/07067437251411023

**Published:** 2026-02-04

**Authors:** Camille Thériault, Lionel Cailhol, Simon Poirier, Félix-Antoine Bérubé, Estelle Ouellet, Pierre David, Alexandre Hudon

**Affiliations:** 1 Département de psychiatrie et d’addictologie, Faculté de médecine, 5622 Université de Montréal, Montreal, QC, Canada; 2 Département de psychiatrie, 26612 Institut universitaire en santé mentale de Montréal, Montreal, QC, Canada; 3 514989 Centre de recherche de l’Institut universitaire en santé mentale de Montréal, Montreal, QC, Canada; 4 D épartement de psychiatrie, Institut national de psychiatrie légale Philippe-Pinel, Montreal, QC, Canada

**Keywords:** trouble de la personnalité limite, médias sociaux, TikTok, analyse qualitative, stigmatisation sociale, relations interpersonnelles, santé mentale, mésinformation, diagnostic psychiatrique, isolement social, borderline personality disorder, social media, TikTok, qualitative analysis, social stigma, social stigmatisation, social stigmatisation, interpersonal relations, interpersonal relationships, mental health, disinformation, psychiatric diagnosis, social isolation

## Abstract

**Objectif:**

À l’ère des réseaux sociaux, la perception publique du trouble de la personnalité limite (TPL) se construit autant dans les espaces cliniques que dans les commentaires en ligne. Cette étude visait à explorer la manière dont les internautes réagissent aux vidéos TikTok portant sur le TPL. L’objectif principal était d’identifier les thématiques émergentes dans les commentaires et de mieux comprendre les perceptions sociales du TPL dans l’espace numérique.

**Méthode:**

Une étude qualitative a été réalisée à partir d’une collecte systématique de 25 197 commentaires issus de 141 vidéos TikTok publics portant sur le TPL. Un échantillon représentatif de commentaires a été extrait et soumis à une analyse thématique, selon une méthode d’itération catégorielle. Les thèmes et sous-thèmes ont été dégagés de manière inductive, à partir d’un codage rigoureux et d’une validation croisée entre évaluateurs.

**Résultats:**

Les résultats révèlent une forte préoccupation des internautes concernant la confusion diagnostique entre le TPL et d’autres troubles psychiatriques, notamment le trouble du spectre de l’autisme et le TDAH. La question de l’auto-diagnostic et de l'auto-traitement est fréquemment abordée, traduisant un besoin d'appropriation identitaire et une méfiance envers les diagnostics professionnels. L’impact du TPL sur les relations interpersonnelles est largement évoqué, oscillant entre tensions relationnelles et stratégies d'adaptation. Le rôle ambivalent des réseaux sociaux est mis en évidence, à la fois comme espace de soutien et vecteur de mésinformation. Enfin, les internautes rapportent des expériences d’isolement social, de stigmatisation et de crainte d’être étiquetés, en soulignant l’importance du soutien affectif dans le parcours de rétablissement.

**Conclusions:**

TikTok devient un espace de construction des identités en santé mentale pour les personnes vivant avec un TPL. Nos résultats appellent à développer des initiatives d’éducation numérique et des interventions ciblées pour contrer la stigmatisation et soutenir les trajectoires de soins dans l’environnement numérique.

## Plain Language Summary Title

Ce que TikTok révèle sur le trouble de la personnalité limite : une étude des conversations en ligne autour du vécu, des stéréotypes et du besoin de soutien

**Plain Language Summary:**

Le trouble de la personnalité limite (TPL) est une condition de santé mentale marquée par une grande sensibilité émotionnelle, des relations instables et un profond sentiment de vide. Aujourd'hui, les réseaux sociaux, et particulièrement TikTok, jouent un rôle important dans la manière dont les personnes parlent de leur santé mentale et se reconnaissent dans certains diagnostics.

Cette étude visait à comprendre comment les internautes perçoivent et discutent du TPL sur TikTok. Les chercheurs ont analysé plus de 25 000 commentaires publiés sous 141 vidéos portant sur ce sujet. Ces messages montrent que beaucoup de personnes vivent de la confusion entre le TPL et d'autres troubles comme l'anxiété, le TDAH ou l'autisme. Plusieurs évoquent des expériences d'auto-diagnostic et expriment une méfiance envers les professionnels, tandis que d'autres partagent leurs difficultés dans les relations amoureuses ou familiales.

Les résultats montrent que TikTok est à la fois un lieu de soutien et de mésinformation. Les internautes y trouvent compréhension et communauté, mais aussi des idées parfois erronées sur le TPL. La stigmatisation et la peur d'être jugé demeurent des thèmes très présents, tout comme la recherche de sens et de rétablissement à travers les partages d'expériences.

En somme, TikTok agit comme un miroir social où se construisent des identités liées à la santé mentale. Cette réalité invite les professionnels à mieux comprendre les usages numériques pour accompagner les personnes vivant avec un TPL et lutter contre les préjugés persistants.

## Abstract (en)

**Objective:**

In the age of social media, public perception of borderline personality disorder (BPD) is shaped as much by clinical settings as by online comments. This study aimed to explore how internet users react to TikTok videos about BPD. The primary objective was to identify emerging themes in the comments and to better understand social perceptions of BPD in the digital space.

**Method:**

A qualitative study was conducted, based on the systematic collection of 25,197 comments from 141 public TikTok videos about BPD. A representative sample of comments was extracted and subjected to thematic analysis using a categorical iteration method. Themes and sub-themes were identified inductively, based on rigorous coding and cross-validation among evaluators.

**Results:**

The results reveal a strong concern among internet users regarding diagnostic confusion between BPD and other psychiatric disorders, particularly autism spectrum disorder and ADHD. The issue of self-diagnosis and self-treatment is frequently addressed, reflecting a need for identity appropriation and mistrust of professional diagnoses. The impact of BPD on interpersonal relationships is widely discussed, ranging from relationship tensions to coping strategies. The ambivalent role of social media is highlighted, both as a source of support and a vehicle for misinformation. Finally, internet users report experiences of social isolation, stigmatisation and fear of being labelled, emphasising the importance of emotional support in the recovery process.

**Conclusions:**

TikTok is becoming a space for building mental health identities for people living with BPD. Our findings call for the development of digital education initiatives and targeted interventions to counter stigma and support care pathways in the digital environment.

## Introduction

Le trouble de la personnalité limite (TPL) est un trouble mental complexe, caractérisé par une instabilité marquée des émotions, de l’image de soi et des relations interpersonnelles.^1^ Les personnes atteintes de ce trouble éprouvent des émotions d’une intensité extrême, avec des fluctuations rapides, qu’elles ont souvent beaucoup de difficulté à réguler. Cette dysrégulation émotionnelle peut conduire à des comportements impulsifs, notamment l’automutilation, les prises de risque inconsidérées et les pensées suicidaires, fréquemment déclenchées par une peur intense de l’abandon, réel ou perçu.^1^ À cette instabilité affective s’ajoute souvent un sentiment chronique de vide intérieur, ainsi que des épisodes transitoires de symptômes dissociatifs ou de pensées de type persécutoire, particulièrement en période de stress aigu.^2^

La prévalence du TPL dans la population générale est estimée entre 0,5% et 2,7%, mais elle atteint jusqu’à environ 10% dans les services spécialisés en santé mentale, notamment en psychiatrie ambulatoire, ce qui témoigne de l'impact disproportionné du trouble dans les contextes cliniques.^3^ Outre sa fréquence, le TPL présente une sévérité marquée au niveau fonctionnel ou en termes de réduction de l’espérance de vie, notamment en lien avec le suicide.^4–6^ Malgré sa fréquence et la lourdeur de ses conséquences fonctionnelles, le TPL demeure un trouble souvent négligé, mal compris et fortement stigmatisé, tant dans le milieu médical qu’au sein de la population générale. Cette stigmatisation contribue à l'isolement social des personnes concernées, et peut entraîner des retards significatifs dans l'accès aux soins appropriés, exacerbant ainsi la sévérité de l'évolution clinique.^7^

Parallèlement, les réseaux sociaux sont devenus une source fréquemment sollicitée pour obtenir des informations sur la santé, y compris la santé mentale.^8^ Si leur utilisation dans ce domaine est encore relativement récente sur le plan scientifique, les premières études indiquent que la diffusion de mésinformation est un phénomène courant. Cette mésinformation varie en fonction des troubles psychiques abordés et des traitements évoqués, ce qui soulève des préoccupations majeures en termes de qualité de l'information accessible au grand public.^8^ Néanmoins, les réseaux sociaux offrent aussi des avantages importants : ils facilitent les discussions ouvertes sur les maladies mentales, contribuent à sensibiliser le public, et participent ainsi à la réduction de la stigmatisation entourant ces troubles.^8^ Ils permettent également un accès élargi à des ressources et à des informations utiles, notamment pour des individus qui pourraient autrement rencontrer des obstacles dans les circuits traditionnels de soins. Toutefois, la dissémination massive de contenus erronés ou approximatifs demeure un risque préoccupant, pouvant entretenir des idées fausses ou retarder l'accès à des soins appropriés.^8^

Les vidéos courtes, surtout celles diffusées sur TikTok, sont devenues un moyen privilégié pour obtenir des conseils et partager des expériences en santé mentale. TikTok est une plateforme sociale centrée sur la diffusion de vidéos brèves, caractérisée par un algorithme de recommandation personnalisé favorisant un engagement rapide et viral. Bien que ce format ait été repris sur d’autres plateformes comme Instagram, YouTube et Facebook, TikTok se distingue par ses outils de montage, ses tendances participatives et la forte implication des jeunes adultes, particulièrement concernés par la santé mentale. Il s’agit donc d’un espace numérique où les discussions sur le TPL sont fréquentes, visibles et largement commentées.^9^

Sur ces réseaux, une variété de créateurs de contenu, qu'ils soient professionnels de la santé ou non, publient des témoignages personnels, des conseils pratiques et des informations sur une multitude de troubles psychiques.^10^ Cette dynamique crée un écosystème hybride, entre sensibilisation, soutien par les pairs et parfois mésinformation. Malgré leur popularité croissante et leur potentiel éducatif, ces plateformes demeurent paradoxalement peu exploitées comme outils d'intervention formelle et restent encore insuffisamment étudiées dans les champs de la psychiatrie et de la psychologie scientifique.^11^

La littérature scientifique demeure encore limitée quant à l’exploration des liens spécifiques entre le TPL, l’utilisation des réseaux sociaux, et la propagation de mésinformation, particulièrement sur des plateformes émergentes comme TikTok.^12^ Les données disponibles suggèrent que les personnes présentant des difficultés interpersonnelles marquées, comme celles vivant avec un TPL, recourent fréquemment aux espaces numériques comme canal de recherche de rassurance sociale, notamment dans le but de valider leurs émotions et d’atténuer un sentiment d’insécurité relationnelle. Elles seraient également plus enclines à divulguer des informations personnelles de manière impulsive, exposant ainsi leur vulnérabilité émotionnelle à des audiences parfois imprévisibles.^13^

Les témoignages personnels de personnes atteintes de TPL, largement partagés sur les réseaux sociaux, attirent souvent une attention considérable. Ces partages peuvent avoir des effets bénéfiques importants, en favorisant la sensibilisation du grand public, en renforçant le soutien entre pairs, et en offrant aux professionnels de la santé mentale un aperçu précieux du vécu quotidien des patients en dehors des contextes cliniques traditionnels.^9^ Cependant, ces dynamiques ne sont pas dénuées de risques. Il est essentiel de rester attentif aux dérives possibles, notamment le sensationnalisme ou la diffusion de contenus réducteurs et parfois inexacts.^9^ De telles représentations biaisées peuvent entretenir des stéréotypes néfastes et donner une image simplifiée, voire déformée, de la complexité du TPL. Dans ce contexte, analyser de manière systématique l’engagement du public (mentions, commentaires, partages) et les réactions suscitées par ces vidéos devient une étape essentielle pour mieux comprendre leur impact réel sur la perception sociale du trouble et sur les trajectoires d’aide et de traitement.

L’objectif principal de ce projet de recherche est d’identifier les thématiques présentes dans les commentaires d’utilisateurs réagissant aux vidéos portant sur le trouble de la personnalité limite sur la plateforme TikTok. Nous faisons l’hypothèse que les types d’interactions seront variés, mais principalement centrés sur les enjeux liés au diagnostic et à la stigmatisation, deux thèmes largement discutés sur les réseaux sociaux à l’heure actuelle.

## Méthodologie

Conformément aux Standards for Reporting Qualitative Research (SRQR), cette étude a suivi un devis qualitatif fondé sur une approche inductive, visant à générer une compréhension approfondie des perceptions et réactions du public à l’égard du TPL sur la plateforme TikTok.^14^ L’étude a été conçue pour répondre aux critères de transparence méthodologique, de réflexivité, d’ancrage empirique, et de pertinence sociale, tel que recommandé par le SRQR. Le projet a été mené en trois grandes étapes : sélection des vidéos, collecte des données et analyse thématique.

### Sélection des Vidéos

Les vidéos ont été sélectionnées à partir d’un corpus préexistant issu d’une recherche exploratoire antérieure qui avait colligé 1000 vidéos, publiées entre septembre 2016 et juin 2024, parmi lesquelles il y avait 207 vidéos TikTok portant explicitement sur le TPL.^15^ La sélection initiale des vidéos a été guidée par une stratégie de variation maximale visant à refléter une diversité de créateurs et de points de vue. Ensuite, pour sélectionner les vidéos de la présente étude, les vidéos devaient satisfaire à plusieurs critères d’inclusion : plus de 10 000 vues, plus de 50 commentaires, contenu spécifiquement centré sur le TPL (témoignages, explications, sensibilisation, etc.), et accessibilité publique avec les commentaires activés. Les vidéos issues de comptes privés, corporatifs ou dont les commentaires étaient désactivés ont été exclues. Les vidéos issues de comptes corporatifs ont été exclues afin de privilégier des contenus spontanés et expérientiels, plus représentatifs des voix individuelles des internautes. Les mots-clés utilisés pour repérer le contenu comprenaient notamment : #borderline, #BPD, #mentalhealth, et #borderlinepersonalitydisorder.

### Collecte de Données

La collecte des commentaires a eu lieu entre août et septembre 2024 à l’aide du logiciel TTCommentsExporter, un outil libre d’accès permettant d’extraire automatiquement tous les commentaires publics associés à une vidéo TikTok. Les données ont été sauvegardées dans des fichiers Excel, chaque ligne correspondant à un commentaire unique, accompagné de métadonnées anonymisées (date de publication, nombre de mentions “j’aime”, identifiant alphanumérique non nominatif de l’utilisateur). Seuls les commentaires disponibles publiquement au moment de l’extraction ont été inclus dans l’analyse, conformément aux normes éthiques de la Politique des trois Conseils sur l’éthique de la recherche avec des êtres humains.^16^

### Analyse de Données

Les données ont été analysées selon la méthode de l’itération catégorielle proposée par Miles et al., en cohérence avec les principes du SRQR.^17^ L’unité d’analyse a été le commentaire individuel, considéré comme une unité expressive de signification autonome. Chaque commentaire a été individuellement annoté par AH et CT et révisé ensemble.

L’analyse a été effectuée en trois cycles progressifs :

- Codage ouvert : Chaque commentaire a été codé manuellement de façon inductive, sans grille préétablie, à l’aide du logiciel QDAMiner.^18^ Les codes initiaux ont été générés de manière descriptive pour refléter le contenu explicite et implicite des messages.
- Regroupement thématique : Les codes ont été regroupés en catégories thématiques plus larges, telles que la stigmatisation, la confusion diagnostique, le soutien par les pairs ou encore la dénonciation du système de soins. Des discussions entre membres de l’équipe ont permis d’ajuster et de clarifier la définition des thèmes, favorisant une convergence interprétative. Un même commentaire pouvait être attribué à plusieurs sous-thèmes lorsque son contenu recoupait différentes dimensions analytiques, ce qui a permis de préserver la richesse des données sans restreindre artificiellement leur signification.
- Affinement et théorisation : À la suite de l’atteinte de la saturation thématique, les thèmes ont été consolidés et illustrés par des verbatims significatifs. Des mémo-théoriques ont été rédigés pour documenter les liens entre les catégories pertinentes. Des ajustements ont été apportés de manière itérative jusqu’à l’obtention d’une représentation fidèle et nuancée de la grille finale de codification.

## Résultats

L’analyse thématique des 25 197 commentaires issus des 141 vidéos TikTok, qui répondaient aux critères de sélection, portant sur le TPL, a permis de dégager huit grands thèmes, chacun structuré en sous-thèmes illustrant la diversité des perceptions, des expériences et des enjeux évoqués par les internautes. Pour en arriver à une saturation des données, il a fallu annoter 1592 commentaires. Les résultats sont présentés selon une approche interprétative, en lien avec le contexte numérique dans lequel ces discours émergent. La liste des thèmes et sous thèmes est présentée dans le Tableau 1.

**Tableau 1.** Liste des Thèmes et de Sous-Thèmes Identifiés Ainsi que Leur Description.

Thème	Sous-thème	Description
Chevauchement symptomatique et confusion diagnostique	Confusion diagnostique	Difficulté à distinguer les troubles aux symptômes similaires, comme le TPL, le trouble bipolaire, le trouble du spectre de l’autisme et le TDAH
	Erreurs de classification	Perception d’un changement ou d’une confusion de diagnostic non justifiée.
Diagnostic et identification de soi	Auto-diagnosticet auto-traitement	Tendance à s’auto-diagnostiquer à partir de comportements ou de symptômes perçus.
	Remise en question du diagnostic professionnel	Remise en question ou réinterprétation du diagnostic posé par un professionnel.
Impact sur les relations	Tensions dans les relations proches	Impact négatif du TPL sur les relations amoureuses et familiales.
	Stratégies d’adaptation relationnelles	Moyens utilisés pour maintenir une relation malgré les symptômes du trouble.
Médication et traitement	Rôle de la médication	Utilisation de médicaments pour stabiliser l’humeur ou réduire certains symptômes.
	La thérapie comme outil de transformation	Mention de thérapies, notamment la TCD, comme outil de gestion efficace.
Perception publique et stigmatisation	Stéréotypes et préjugés	Dénonciation des idées reçues véhiculées par la société ou les médias.
	Crainte d’être étiqueté	Peur de porter l’étiquette associée au TPL ou d’être jugé à cause du diagnostic.
Défis émotionnels et comportementaux	Instabilité émotionnelle	Difficultés à réguler les émotions, menant à une souffrance marquée.
	Comportements manipulateurs perçus	Propos sur des comportements perçus comme manipulateurs dans les relations.
Isolement social et soutien	Isolement découlant des symptômes	Sentiment d’isolement social lié au diagnostic ou à ses manifestations.
	Soutien comme facteur protecteur	Importance du soutien familial ou communautaire dans le processus de rétablissement.
Traumatisme et troubles de la personnalité	Traumatisme précoce	Lien fait entre le TPL et des traumatismes vécus durant l’enfance.
	Comportements liés au trauma	Comportements défensifs ou d’évitement en lien avec un passé traumatique.

### Chevauchement Symptomatique et Confusion Diagnostique

Les internautes ont exprimé leur perplexité face à la complexité du TPL notamment en raison du chevauchement symptomatique avec d’autres troubles psychiatriques.

#### Sous-Thème : Confusion Diagnostique

La difficulté à distinguer le TPL d’autres troubles comme l’autisme, le TDAH ou le trouble bipolaire est une préoccupation récurrente. De nombreux internautes rapportent des symptômes similaires (instabilité émotionnelle, impulsivité) rendant ardu un diagnostic clair et stable. Certains évoquent des diagnostics successifs contradictoires, renforçant frustration et perte de confiance envers les professionnels. La circulation d’informations simplifiées sur les réseaux sociaux accentue cette confusion, alimentant amalgames et malentendus.^19^

Verbatim :

« Ma théorie est que presque toutes les personnes TPL ont un Trouble du spectre de l’autisme et/ou un TDAH, combinés avec un TSPT-C, ce qui entraîne certains comportements. S’il existe une distinction entre cela et le TPL, je ne l’ai pas encore observée. »

« Attention, le TPL peut aussi être un trouble bipolaire qui ressemble au TPBL. Assurez-vous de consulter un médecin avant de vous auto-diagnostiquer. »

#### Sous-Thème : Erreurs de Classification

Plusieurs utilisateurs relatent des changements répétés de diagnostic sans explication claire. Ce manque de transparence alimente la méfiance et complique l'engagement thérapeutique. Les erreurs de classification peuvent mener à des interventions inadaptées et nourrir l'instabilité identitaire déjà fragilisée chez les personnes atteintes de TPL.^20^

Verbatim :

« Je me demande pourquoi on a changé mon diagnostic de TPL pour autre chose … »

« C'était tellement utile. Moi qui passe mon temps à m'inquiéter : “Et si c'était moi, la personne narcissique ?” Élevée par un(e) narcissique. Diagnostiquée à tort comme bipolaire, en cours d’évaluation pour l’autisme, je me reconnais énormément dans le contenu sur le trouble borderline. »

### Diagnostic et Identification de soi

Les commentaires révèlent une tension entre l'identification professionnelle et l'auto-diagnostic.

#### Sous-Thème : L’auto-Diagnostic et L’auto-Traitement

Face à des vécus émotionnels intenses, beaucoup tentent de s’auto-diagnostiquer, influencés par des témoignages en ligne ou par l'observation d'effets médicamenteux. Si cette quête de compréhension est légitime, elle comporte des risques : confusion entre TPL, trouble bipolaire ou anxiété sévère, et adoption de stratégies d’auto-traitement parfois inadaptées, aggravant les symptômes.^21^

Verbatim :

« Peut-être que je devrais reprendre mon antipsychotique … »

« Mais je pense que je pourrais aussi avoir un trouble de la personnalité limite »

#### Sous-Thème : Remise en Question du Diagnostic Professionnel

Certains proches questionnent un diagnostic ou expriment leur scepticisme envers le diagnostic reçu par un membre de leur famille, pointant un possible surdiagnostic ou une compréhension incomplète de la situation. Il est possible que l’absence de justification clinique claire, illustrée par certains propos, puissecontribuer à fragiliser la confiance dans le processus thérapeutique, bien que cette dimension n’ait pas été formulée explicitement par les internautes.

Verbatim :

« Le psychiatre a dit TPL, mais je pense que c’est un TDAH. »

« Après avoir reçu le diagnostic de borderline, je ne suis pas convaincue … ça ne correspond pas à ce que je vis »

### Impact sur les Relations

Le TPL a un impact profond sur les dynamiques relationnelles, générant à la fois tensions et stratégies d’adaptation.

#### Sous-Thème : Tensions Dans les Relations Avec les Proches

L’instabilité émotionnelle rend les relations imprévisibles. Les proches parlent d'un sentiment de « marcher sur des œufs », craignant des réactions émotionnelles disproportionnées. Les cycles d’idéalisation et de rejet renforcent l'insécurité émotionnelle des deux côtés.

Verbatim :

« Mon mari a été diagnostiqué TPL, et depuis, notre relation est devenue très difficile. »

« C’est vraiment dur de vivre avec quelqu’un qui est constamment en montagne russe … »

#### Sous-Thème : Stratégies D’adaptation Relationnelles

Pour faire face, plusieurs évoquent des stratégies comme la thérapie individuelle, les groupes de soutien ou la prise de distance émotionnelle temporaire. Ces approches permettent de préserver le lien tout en protégeant leur bien-être émotionnel.^22^ Dans certains cas, les internautes ne décrivent pas tant des stratégies activement mises en place que la présence d’un partenaire soutenant, jouant un rôle protecteur face à l’instabilité émotionnelle.

Verbatim :

« Quand j’ai eu mes propres enfants, je n’ai pas réalisé à quel point une grande partie de cette relation était émotionnellement destructrice et inappropriée. J’ai établi une limite pour me protéger, moi et mes enfants. »

« Pour moi, mon petit ami est simplement patient, parle lentement et apprend mon langage de l’amour comme je l’ai dit. Ça aide aussi à stabiliser mes humeurs. Tout repose sur le calme, l’écoute, et à quel point on se sent vu et compris »

### Médication et Traitement

Le rôle de la médication et de la psychothérapie dans le TPL est largement discuté.

#### Sous-Thème : Rôle de la Médication

La médication est perçue comme un outil d’apaisement émotionnel ponctuel, mais son effet limité sur les enjeux relationnels et identitaires est souligné. Les témoignages rapportent aussi une ambivalence face aux effets secondaires.

Verbatim :

« Ma médication m’aide à me calmer quand je sens que je perds le contrôle. »

« Il n’existe pas de remède, cela peut être “apaisé” avec des médicaments, mais même dans ce cas, on en souffre toujours, ça fait juste retomber la pression. »

#### Sous-Thème : La Thérapie Comme Outil de Transformation

La thérapie, en particulier la thérapie comportementale dialectique (TCD), est décrite comme une ressource transformatrice. L'apprentissage de la pleine conscience, de la tolérance à la détresse et de l'efficacité interpersonnelle est valorisé comme un levier pour un changement en profondeur.

Verbatim :

« La TCD m’a vraiment aidé à reprendre le contrôle sur mes réactions. »

« J’ai reçu un diagnostic de TPL et la thérapie, en particulier TDC, peut aider. La spiritualité m’a également beaucoup aidé. »

### Perception Publique et Stigmatisation

Les discours révèlent une tension entre désir de reconnaissance et crainte du rejet social.

#### Sous-Thème : Stéréotypes et Préjugés

De nombreux internautes dénoncent les stéréotypes caricaturaux véhiculés par les médias et par les cliniciens, associant le TPL à la dangerosité ou à la manipulation. Cette stigmatisation freine l’accès aux soins et renforce l'isolement social.

Verbatim :

« Les gens pensent toujours qu’on est dangereux ou instables, c’est faux et blessant. »

« Je suis là si quelqu’un cherche un ami. Ce n’est pas parce que j’ai un TPL que je suis une personne horrible. En fait, je suis vraiment quelqu’un de gentil. »

#### Sous-Thème : Crainte D’être étiqueté

La peur d’être réduit à son diagnostic est omniprésente. Plusieurs internautes expriment leur hésitation à chercher une aide professionnelle ou à divulguer leur trouble, par crainte du jugement et de la discrimination.

Verbatim :

« Je ne veux pas qu’on me colle l’étiquette de TPL, c’est comme un fardeau. »

« Je ne veux pas recevoir de diagnostic ni avoir un trouble de la personnalité, parce que ça peut te suivre toute ta vie. »

### Défis Émotionnels et Comportementaux

Les internautes décrivent leurs luttes émotionnelles de manière introspective.

#### Sous-Thème : Instabilité émotionnelle

L'instabilité émotionnelle est perçue comme un combat quotidien : des changements d’humeur rapides, des pertes de contrôle émotionnel, et une incapacité à stabiliser les relations.

Verbatim :

« Parfois, mes émotions prennent le dessus, je n’arrive pas à me calmer. »

« La rage intérieure est vraiment intense. Tu l’as parfaitement dit : de 0 à 100 en une seconde !!! »

#### Sous-Thème : Comportements Manipulateurs Perçus

Des proches perçoivent certains comportements comme manipulateurs, bien que ceux-ci soient souvent le reflet d'une détresse extrême et d'une peur d'abandon. Le malentendu entre intention vécue et perception externe renforce les tensions relationnelles.^23^

Verbatim :

« Il manipulait tout le monde autour de lui, c’est lié à son trouble. »

« Mon entourage pense que je fais du chantage, alors que je veux juste qu’on reste avec moi. »

### Isolement Social et Soutien

Isolement et recherche de soutien sont des dimensions centrales du vécu partagé.

#### Sous-Thème : Isolement Découlant des Symptômes

L'instabilité émotionnelle et l’impulsivité contribuent à l’érosion des liens sociaux, menant à un isolement parfois choisi, mais douloureux, renforçant le cercle vicieux de la détresse émotionnelle.^24^

Verbatim :

« C’est trop dur de garder des amis quand on a un TPL … »

« Pareil … c’est comme si soit personne ne voulait comprendre, soit quand ils essaient, je les repousse exprès. »

#### Sous-Thème : Soutien Comme Facteur Protecteur

Le soutien empathique d’amis, de la famille ou de partenaires ainsi que celui trouvé sur les réseaux sociaux, est décrit comme un facteur de protection important pour faire face aux crises et favoriser l’engagement thérapeutique.

Verbatim :

« Ma famille a été mon pilier tout au long de mon traitement. »

« I've noticed this, and I can feel your energy. I want to be better at comforting you when it happens. »

### Traumatisme et Troubles de la Personnalité

Les liens entre traumatismes précoces et développement du TPL sont largement évoqués.

#### Sous-Thème: Traumatisme Précoce

De nombreux témoignages associent le TPL à des expériences d'abus, de négligence ou d'instabilité affective durant l'enfance, perçues comme des racines profondes des difficultés émotionnelles et relationnelles actuelles.

Verbatim :

« Tout remonte à mon enfance. J’ai vécu des choses très dures. »

« Mon trouble borderline est clairement apparu avant les abus sexuels, mais je ne me souviens pas de la majeure partie de ma vie, alors qui sait ce qui s’est passé. »

#### Sous-Thème : Comportements Liés au Trauma

Les comportements relationnels problématiques (évitement, méfiance, dépendance affective) sont interprétés comme des réponses automatiques à ces traumatismes précoces.

Verbatim :

« Je repousse tout le monde, c’est à cause de mon passé. »

« Le côté dissociatif est lié aux traumatismes vécus et rejoués inconsciemment. »

## Discussion

Le présent projet visait à explorer les perceptions et expériences exprimées par des internautes en réaction à des vidéos TikTok portant sur le TPL. L’analyse thématique des commentaires a permis de dégager plusieurs thèmes centraux, notamment la confusion diagnostique, la stigmatisation, le rôle du soutien social, l'impact des traumatismes précoces, ainsi que l'utilisation des médias sociaux pour l'auto-diagnostic et la recherche de validation. À travers ces discours, se dessine une tension constante entre la recherche de compréhension, la souffrance émotionnelle et les enjeux d’appartenance sociale.

Un premier concept particulièrement saillant est celui du chevauchement symptomatique entre le TPL et d’autres troubles, en particulier le trouble du spectre de l’autisme, le TDAH et le trouble bipolaire. Ce phénomène est bien documenté dans la littérature scientifique pour le trouble bipolaire, où plusieurs études soulignent que l'instabilité de l'humeur dans le TPL peut être confondue avec les épisodes thymiques du trouble bipolaire, contribuant à des erreurs diagnostiques fréquentes.^25,26^ Cette confusion est d'autant plus problématique que les traitements optimaux diffèrent radicalement selon le trouble identifié. Nos résultats rejoignent ainsi les constats cliniques récents, qui appellent à une évaluation longitudinale et nuancée des troubles de l’affect chez les jeunes adultes pour limiter les erreurs de classification.^27^

Un deuxième concept concerne l’auto-diagnostic et l’appropriation identitaire numérique, particulièrement chez les jeunes. Naslund et al. (2020) ont montré que les réseaux sociaux, notamment TikTok, favorisent une « vulgarisation émotionnelle » des diagnostics psychiatriques, réduisant des troubles complexes à des listes simplifiées de symptômes.^28^ Cette simplification répond à un besoin légitime : expliquer le trouble à partir d’une expérience vécue, dans un langage accessible et incarné. Pour plusieurs, ces contenus servent de miroir identitaire, mettant en mots des états longtemps confus ou invalidés. Ce processus, s’il favorise reconnaissance de soi et sentiment de communauté, comporte aussi des risques : sans accompagnement clinique, il peut encourager surdiagnostics, rigidifier des identités psychopathologiques ou retarder l’accès à des soins. Il importe donc de comprendre ce double mouvement entre appropriation narrative et simplification médicale afin d’articuler les savoirs expérientiels numériques aux cadres diagnostiques professionnels.

Un troisième axe de convergence concerne l’impact des traumatismes précoces sur la trajectoire du TPL, largement évoqué dans les commentaires et confirmé par la littérature. Une méta-analyse récente montre que les abus émotionnels et physiques durant l’enfance augmentent significativement le risque de traits de TPL à l’âge adulte. Nos résultats indiquent que cette origine traumatique est intégrée dans la narration des personnes concernées : les blessures d’attachement influencent encore leurs relations et leur régulation émotionnelle.^29^ Sur les réseaux sociaux, cette mise en récit contribue à redéfinir le TPL, non plus comme une « tare personnelle », mais comme le produit d’événements adverses. Ces récits déplacent ainsi le blâme historiquement associé au TPL, soulignant ses origines intersubjectives et environnementales. Ce changement restaure la dignité tout en dénonçant l’injustice liée à la pauvreté des services et à une approche responsabilisante, souvent vécue comme une mise en cause personnelle ou mal comprise en clinique. Les plateformes comme TikTok deviennent alors des espaces de réparation narrative et de recomposition identitaire hors des cadres médicaux traditionnels.

Malgré la richesse des données recueillies, ce projet présente plusieurs limites. D'abord, l'analyse repose sur des commentaires publics, ce qui introduit un biais de sélection, car les individus les plus vulnérables ou ceux ayant une approche plus critique des réseaux sociaux pourraient être sous-représentés. Les individus vivant une détresse psychologique plus sévère ou un isolement marqué pourraient être sous-représentés dans notre corpus, car ils participent possiblement moins souvent aux espaces publics en ligne ou choisissent de s’exprimer dans des contextes plus privés. De plus, les interprétations doivent être nuancées par le fait que les commentaires sont souvent brefs et contextuels, ne permettant pas toujours d’accéder à la complexité du vécu émotionnel des utilisateurs. Enfin, notre analyse s’est limitée à TikTok et aux utilisateurs qui choisissent de s’exprimer publiquement. Les dynamiques pourraient différer sur d’autres plateformes sociales (p. ex. Instagram, YouTube) ou chez les usagers silencieux, absents des espaces visibles. Ces angles constituent des pistes de recherche futures pour mieux comprendre la diversité des expériences numériques.

## Conclusions

Ce projet, fondé sur l’analyse de commentaires TikTok, éclaire comment les internautes perçoivent, interprètent et partagent leur expérience du TPL. Les récits expriment une quête de sens face à la souffrance, une réappropriation identitaire et une remise en question des étiquettes diagnostiques.

TikTok apparaît comme un espace d’expression et de transformation identitaire, contrastant avec un système médical où la parole des personnes TPL demeure limitée. Ces plateformes permettent de rendre visible le vécu, d’obtenir reconnaissance et de contester la stigmatisation.

Ces dynamiques incitent les milieux cliniques à voir les réseaux sociaux comme vecteurs d’intervention. En utilisant leurs codes visuels et narratifs, les professionnels pourraient diffuser des contenus probants et contrer la mésinformation. Mieux comprendre ces récits, c’est contribuer à une psychiatrie plus humaine, accessible et ancrée dans les réalités actuelles.
